# Quantum nanoconstrictions fabricated by cryo-etching in encapsulated graphene

**DOI:** 10.1038/s41598-019-50098-z

**Published:** 2019-09-19

**Authors:** V. Clericò, J. A. Delgado-Notario, M. Saiz-Bretín, A. V. Malyshev, Y. M. Meziani, P. Hidalgo, B. Méndez, M. Amado, F. Domínguez-Adame, E. Diez

**Affiliations:** 1 Group of Nanotechnology, USAL-NANOLAB, Universidad de Salamanca, E-37008 Salamanca, Spain; 2 Departamento de Física de Materiales, Universidad Complutense, E-28040 Madrid, Spain; 3 Ioffe Physical-Technical Institute, 26 Politechnicheskaya str., 194021 St. Petersburg, Russia

**Keywords:** Electronic properties and devices, Condensed-matter physics

## Abstract

We report on a novel implementation of the cryo-etching method, which enabled us to fabricate low-roughness hBN-encapsulated graphene nanoconstrictions with unprecedented control of the structure edges; the typical edge roughness is on the order of a few nanometers. We characterized the system by atomic force microscopy and used the measured parameters of the edge geometry in numerical simulations of the system conductance, which agree quantitatively with our low temperature transport measurements. The quality of our devices is confirmed by the observation of well defined quantized 2*e*^2^/*h* conductance steps at zero magnetic field. To the best of our knowledge, such an observation reports the clearest conductance quantization in physically etched graphene nanoconstrictions. The fabrication of such high quality systems and the scalability of the cryo-etching method opens a novel promising possibility of producing more complex truly-ballistic devices based on graphene.

## Introduction

Graphene stands out as one of the most promising 2D materials for electronic applications. Initial problems related to the low charge carrier mobility, which were typical for the first standalone graphene samples, have been successfully resolved. For example, it has been demonstrated that the charge mobility can increase by several orders of magnitude by encapsulating graphene between thin layers of diverse 2D materials, such as hexaboron-nitride (hBN)^1^. It has been demonstrated that the charge mobility can increase by several orders of magnitude in these structures. However, a fully-tunable electronic confinement of charge carriers in graphene, that has been pursued since its discovery^2^, is still remaining challenging. It turned out that edge imperfections of graphene based systems are so detrimental for the ballistic charge transport that observations of the quantized conductance even in the simplest nanostructures, such as a nanoconstriction (NC), was problematic.

Up to now, manifestations of conductance quantization have been observed in nanoscopic channels created by either electrostatic gating or by methods that change the geometry of the sample, such as mechanical or chemical etching. While the former electrostatic approach showed promising results with well defined 2*e*^2^/*h* or 4*e*^2^/*h* spaced conductance plateaus^3,4^, the latter has turned into an arduous technological challenge and yielded less clear evidence of quantized conductance. For example, the first mechanically defined NCs were made of graphene deposited directly onto silicon dioxide wafers^2,5^. Conductance steps in those systems appeared to be at least an order of magnitude lower than the conductance quantum 2*e*^2^/*h*^6,7^, which could be caused by a plethora of different effects such as the Coulomb blockade, high electron back scattering and localization by disorder or charging effects, or by intrinsically low mobility of carriers. Recently, Tombros *et al*.^8^ observed 2*e*^2^/*h* steps of conductance in a high mobility constriction produced by current annealing of suspended graphene flakes. The nanostructure was fabricated by passing high electric current through a suspended graphene flake which was deforming into a NC-like structure. The main limitation of the method is purely technological: accurate control of the dimensions of the graphene NC is difficult to attain and, therefore, such nanostructures are not easily reproducible.

On the other hand, encapsulation of graphene between lattice-matched hBN allowed for the fabrication of reproducible high mobility devices^9^. However, the conductance quatization in such devices based on intercalated graphene was reported to manifest itself in the form of *kinks*^10,11^. Well-defined conductance plateaus could not be observed because of the roughness of the heterostructure’s edges and the planar structure itself (the latter resulting from the intrinsic dielectric behavior of the hBN films). These problems were partially overcome by fabricating NCs from exfoliated graphene on hydrophobic silicon oxide substrates with the use of hexamethyldisilazane (HMDS)^12,13^. This method led to a substantial increase of the charge mobility which also remained high for a long period of time (~96 h). Quantized conductance at zero magnetic field was also observed in refs^12,13^, but only in the form of *kinks*, suggesting that the edge definition and roughness control was not sufficiently high. In this contribution we propose a novel cryo-etching method theat enables us to overcome the above mentioned problems and fabricate nanoconstrictions with both very high charge carrier mobility and unprecedented quality of the structure edges.

## Results and Discussion

In this work we present the fabrication method and characterization of NCs on quasi-2D encapsulated graphene where a cryo-etching technique, commonly applied for bulk three-dimensional materials, has been used to achieve an unprecedented control of the edge-definition. This technique was already successfully implemented in silicon-based devices, where it was possible to control and define low-roughness sidewalls, observe the absence of sidewall scalloping^14^, and generate samples with a high aspect ratio and cleaning^15^ in nanometric-size structures^16^. The extension of the cryo-etching technique to graphene allowed us to create NCs with very low sidewall roughness, as proved by atomic force microscopy (AFM) measurements. AFM micrographs were employed to assess the profile of the sample edges, that were later used as input in numerical simulations. We found an excellent agreement between the measured and calculated electrical conductivity and a good correspondence to the theoretical prediction.

We focus the attention on a hBN/graphene/hBN constriction of a few hundreds of width. The constrictions were defined using the innovative cryo-etching process in graphene-based structures in the third aligned EBL step (see Methods and supplementary information S2). The use of a polymethylmethacrylate (PMMA) mask for this etching process represents a less invasive approach for encapsulated graphene-structures compared to the use of a Cr mask in submicron-size constrictions^10^. To the best of our knowledge, this is the first time that a cryo-etching method is successfully used for the definition of graphene nanostructures.

**Figure 1:**
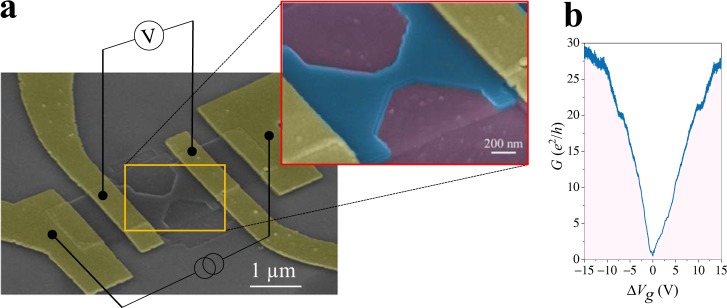
(**a**) Tilted SEM micrograph of an encapsulated graphene NC with lateral width *W* ≃ 206 nm and length *L* ≃ 200 nm including an schematic view of the electronic setup. The inset shows an enlarged view where the hBN/graphene/hBN heterostructure is colored in blue, the SiO_2_ substrate (partially etched) in violet and the contacts in yellow. (**b**) Conductance as a function of the normalized voltage $\Delta {V}_{g}={V}_{g}-{V}_{g}^{\ast }$ for the same NC measured at *T* = 3.1K.

Figure 1a shows a tilted SEM micrograph of a typical NC in encapsulated graphene defined by cryo-etching. The lateral width of this NC is *W* ≃ 206 nm and the length is *L* ≃ 200 nm. The inset shows the SEM micrograph at higher magnification, in which the sandwiched structure (hBN/graphene/hBN) is colored in blue. A standard 4–probe configuration to perform the electronic characterization at low-temperatures is sketched in the figure, where a pseudo-dc (13) current of 5 nA was injected/collected from the outer contacts while the voltage drop was measured by an in-phase lock-in preamplifier closer to the constriction (the contacts are colored yellow for clarity). The result of a typical measurement at *T* = 3.1K is depicted in Fig. 1b. The conductance in units of *e*^2^/*h* is displayed as a function of the normalized back-gate voltage $\Delta {V}_{g}={V}_{g}-{V}_{g}^{\ast }$, where ${V}_{g}^{\ast }$ is the voltage at the Dirac point. The conductance is directly obtained by the measured contactless resistance as *G* = 1/*R*. In our sample the hole-side region (Δ*V*_*g*_ < 0V) is always better resolved than the electron one (Δ*V*_*g*_ > 0V), where the ballistic behaviour starts at higher back-gate voltage due to the residual charge density. For this reason, in the main text we restrict our discussion to the hole-side (see supplementary information S4 for the electron-side regime). The typical mobility in our devices is *μ* ≃ 150000 cm^2^/Vs at room temperature, as shown in the supplementary information S3, obtained by means of the field effect formula^17^.

**Figure 2:**
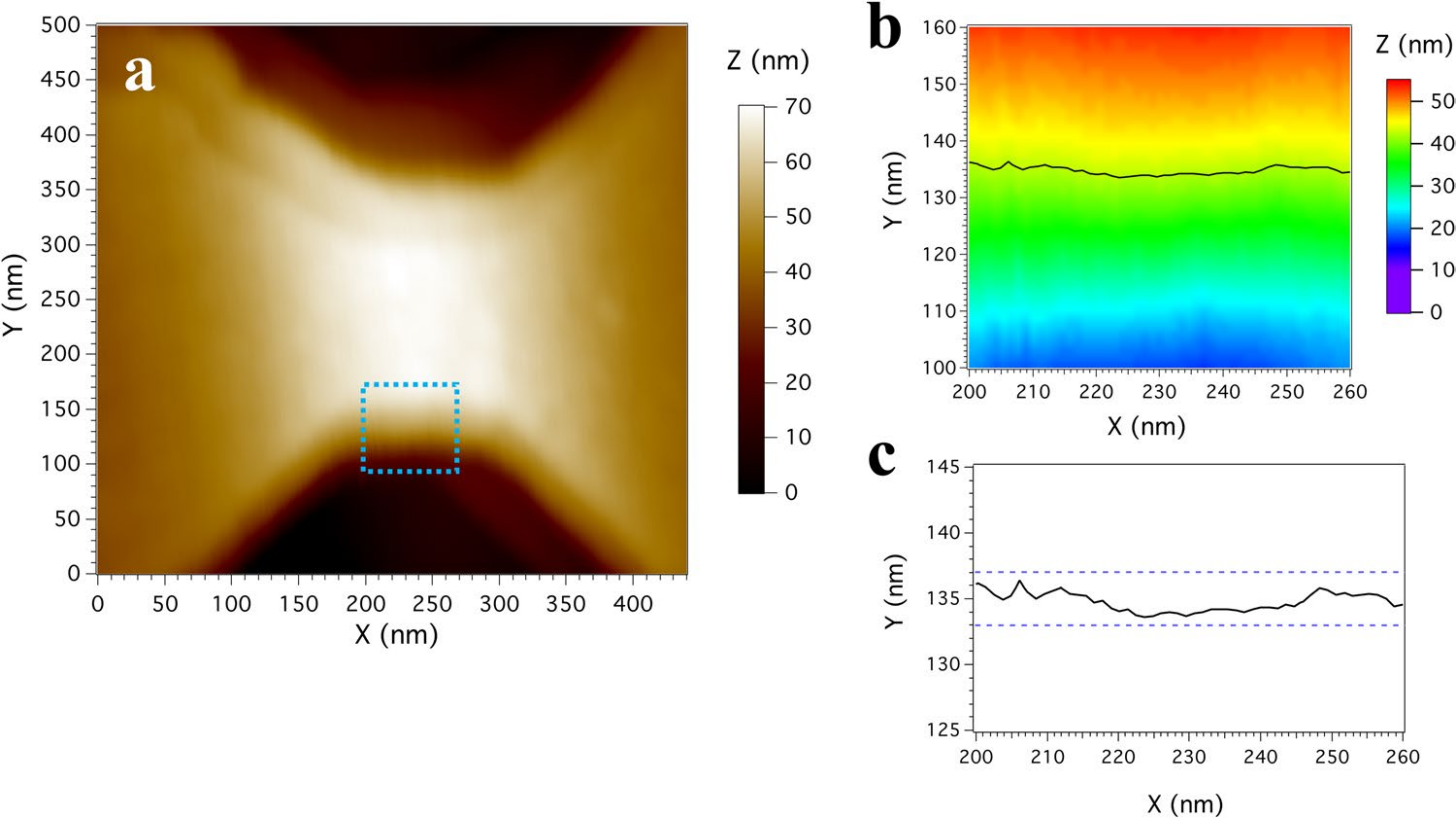
(**a**) AFM image of the graphene NC with *W* = 206 nm. (**b**) Contour plot taken from the square highlighted in panel (a), at 15 nm from the top of the nanostructure. (**c**) Enlarged view of the contour plot, where the dashed lines indicate the values used to estimate the edge roughness.

The quality of the graphene NCs is apparent in the SEM micrograph shown in Fig. 1a. However, in order to get a quantitative analysis of the edge roughness, AFM measurements were performed in our samples. We have used a Nanotech AFM instrument operating in contact mode. Figure 2a displays the AFM image of the whole NC. The edge and the corresponding contour profile within the square marked in panel (a) are shown in Fig. 2b. The AFM images reveal the excellent definition of our graphene NC and, in particular, the smooth sidewall. The contour plot (black line in Fig. 2b) is taken 15 nm under the top hBN flake (with a thickness of about 15 nm) in order to assess the actual size and roughness of the NC. The average edge roughness can be estimated by zooming in this profile and then a roughness around 2 nm is obtained (see Fig. 2c). This value is not far from the edge roughness estimated in lower mobility graphene NCs on HMDS^12^, namely without hBN, where the value was of the order of 1 nm. Our measurement represents the first quantitative estimation of the roughness observed in encapsulated graphene nanoconstrictions.

**Figure 3:**
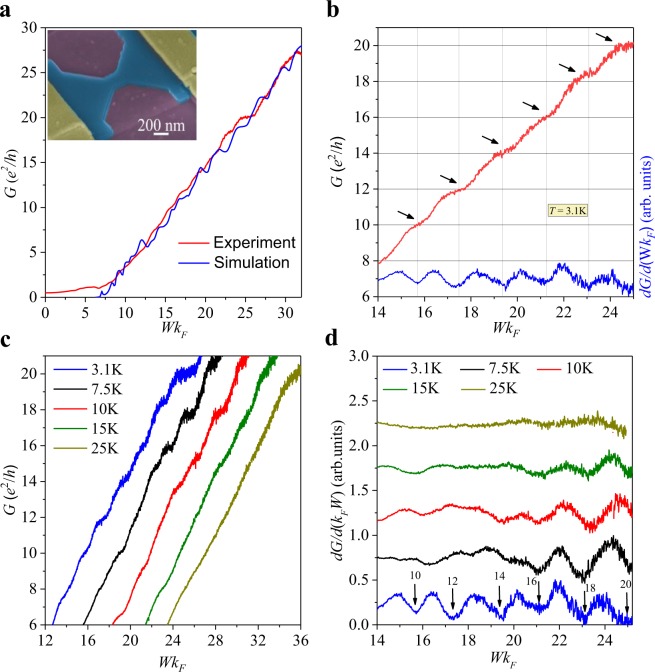
(**a**) Comparison of simulated and experimental conductance of a GNC of width *W* = 206 nm. (**b**) Conductance (red line) and transconductance (blue line) as a function *Wk*_*F*_ measured at *T* = 3.1K. Solid black arrows show the position of the plateaus of conductance separated by 2*e*^2^/*h* and matching integer values from *G* = 10*e*^2^/*h* onwards. (**c**) Evolution of *G* as a function of *Wk*_*F*_ at different temperatures. The curves have been horizontally shifted by a factor 2.5*Wk*_*F*_ for clarity. (**d**) Temperature dependence of the transconductance as a function of *Wk*_*F*_. Black solid arrows represent the value of the plateaus of conductance in units of *e*^2^/*h* at *T* = 3.1K, matching the position of the minima in *dG*/*d*(*Wk*_*F*_).

Thanks to the accurate profile obtained with AFM measurements, we are able to study the real edge roughness in our simulations based on a tight binding model (see Methods for details). Figure 3a shows the conductance *G* as a function of *Wk*_*F*_ (with *k*_*F*_ the Fermi wavenumber) measured at *T* = 3.1K (red line) and the conductance obtained from our tight-binding simulations (blue line) with the same edge roughness obtained from the AFM measurements (see Fig. 2).

In the low-temperature regime, when the elastic mean free path *l*_*m*_ is larger than the GNC length *L* = 206 nm, transport is ballistic. We have estimated from the carrier mobility of our GNC that *l*_*m*_ > 1 μm (see supplementary information S3) in the temperature range considered in this work. Hence conditions for ballistic transport are largely satisfied and therefore the conductance should be also described by the commonly used Sharwin formula^10,18–20^ (replacing the usual factor 2 by a factor 4 to account for both spin and valley degeneracies in graphene) showing a linear dependence of *Wk*_*F*_

1 $$G=\frac{4{e}^{2}}{\pi h}W{k}_{F}t\,,$$

where *t* is the transmission parameter, being *t* = 1 for the ideal ballistic transport.

We notice the good agreement between the experimental and simulated conductance as well as the linear dependence on *Wk*_*F*_ above a threshold value $W{k}_{F}^{0}$, characteristic of ballistic transport. Additionally, the simulated conductance shows small resonant peaks that can be attributed to Fabry-Perot oscillations^21^, while the experimental conductance increases monotonically because temperature is not low enough to reveal this phenomenon. From the normalized gate voltage Δ*V*_*g*_ we can obtain the carrier density *n* and therefore the Fermi wavenumber ${k}_{F}=\sqrt{\pi n}$. Due to the presence of residual doping in our NC, we cannot tune the carrier density below a minimum value *n*_0_ ~ 3.3 × 10^10^ cm^−2^ experimentally, and accordingly *Wk*_*F*_ could not be tuned below a threshold value $W{k}_{F}^{0}=\sqrt{\pi {n}_{0}}\,W \sim 6.7$. The transmission parameter *t* is estimated from the linear fit of the conductance when $W{k}_{F} > W{k}_{F}^{0}$ using Eq. (1). It is worth stressing the remarkable agreement of the slopes obtained from the theoretical and experimental conductance curves, yielding the transmission parameter *t* ~ 0.9. This value is close to the fully transmitted carrier regime *t* = 1, in contrast to the estimation *t*~0.6 given in ref.^10^ for a graphene NC with a comparable width *W* = 230 nm that was not defined by cryo-etching. For the electron-side (${V}_{g} > {V}_{g}^{\ast }$), we also found a very similar transmission parameter *t* ~ 0.9, as presented in the supplemental information S4. This result, supported by the remarkable agreement with theoretical calculations using realistic profiles of the edge roughness, is a clear indication of ballistic transport due to the very high mobility and smooth edges that arise from the novel use of the cryo-etching procedure in their preparation. The estimated value of *t* in our structure is also significantly higher than the one obtained in much narrower NCs of width *W* = 100 nm produced by means of a HMDS treatment^12,13^. In these samples, low-roughness edges arise mainly due to the fact that the etching process on a single layer graphene is more controllable and a clean edge on a single layer of material is easier to attain than the one obtained on a thicker encapsulated graphene nanostructure. The study of graphene NCs obtained by means of a HMDS treatment is also presented in the supplementary information for comparison (S5 and S6). In that case, the corresponding transmission parameters are significantly lower than the ones found in encapsulated graphene NCs defined by cryo-etching.

A closer inspection of the conductance *G* (red line) and the dimensionless transconductance *dG*/*d*(*Wk*_*F*_) (blue line) versus *Wk*_*F*_ are shown in Fig. 3b, signaling the second goal of this work. The plateaus of quantized conductance are clearly observed at regular intervals of *Wk*_*F*_, corresponding to equally-spaced minima in the transconductance. The quantization of *G* is also very clear after reverting the direction of the current (see supplementary information S4). Up to six plateaus appear at values of the conductance of *G* = 10, 12, 14, 16, 18, 20 *e*^2^/*h*, hence the jump between neighbouring plateaus has been found to be 2*e*^2^/*h*. This value matches the theoretical estimation by Guimarães *et al*., where the conductance of a graphene NC where *W*~*L* is quantized into well-defined plateaus separated by 2*e*^2^/*h*^21^. Ihnatsenka *et al*. put forward electron-electron interaction and boundary scattering as the major responsible for the appearance of plateaus separated by 2*e*^2^/*h* instead of 4*e*^2^/*h*^22^, as it would be expected in ideal nanoribbons with perfectly smooth edges where both spin and valley degeneracy are preserved^3^. To the best of our knowledge, this is the largest number of plateaus of conductance observed in single layer graphene samples that have been physically narrowed down into nanoconstriction-shape to date. In contrast, in bilayer graphene it is easier to confine carriers due to the presence of a gap, hence plateaus of conductance are easier to achieve^4,23^. Furthermore, constricting the conductive region electrostatically allows one to narrow the constriction down to tens of nm^3,4^, thus decreasing the number of channels responsible for electric conduction. Our approach becomes a good compromise to achieve almost fully transmitted structures with smooth sidewalls where a systematic experimental study of NCs having differing widths may developed. In Fig. 3c we present the temperature dependence of the conductance (taken at *T* = 3.1, 7.5, 10, 15 and 25K), where the curves have been horizontally shifted by a factor of 2.5*Wk*_*F*_ for clarity. The presence of the plateaus is clear and they are noticeably visible below *T* ~ 10K. They start to smear out at higher temperatures until they are completely washed out at *T* = 25K. Such a temperature dependence of *G* can be better studied by inspecting the dimensionless transconductance as presented in Fig. 3d. The amplitude of the periodic oscillations of the transconductance as a function of *Wk*_*F*_ decreases by increasing temperature and finally disappears above *T* = 15K. A similar threshold temperature for the vanishing of the conductance quantization was very recently found in bilayer graphene constrictions^23^. This behavior differs markedly from other non-desired effects in diffusive regime, such as localized charge carriers, that disappear at higher temperature or other effects that appear also in ballistic graphene quantum point contacts as Fabry-Pérot oscillations^24^.

In summary, we have proposed a novel implementation of the cryo-etching method, which we used for fabrication of high quality hBN-encapsulated graphene nanoconstrictions. The main advantage of the method is an unprecedented control of the edge definition and reduced roughness. The latter enabled us to fabricate high-mobility graphene nanoconstrictions with well defined, sharp and extraordinarily smooth edges. These systems have been characterized by the standard AFM imaging techniques and then the obtained data were used in large scale transport simulations. Results of such modelling agree very well with our experimental low-temperature measurements and suggest that the transport in the system is almost fully ballistic. In particular, both the predicted and measured values of the transmission parameter are as high as *t*~0.9 and the size quantization is clearly manifesting itself as a ladder of several 2*e*^2^/*h*-spaced plateaus of conductance at temperatures up to *T* = 10K, confirming the high quality of the samples. On the other hand, our fabrication method also proves to be scalable. Nanoconstrictions are the simplest model systems which can be viewed as building blocks for more sophisticated devices. We argue therefore that the proposed cryo-etching technique paves the way for the fabrication of more complex graphene-based nanostructured prototypical devices with truly ballistic transport of high-mobility carriers.

## Methods

### Graphene and hBN exfoliation

The fabrication process started from the exfoliation of graphene and hBN on 290 nm silicon oxide thermally grown on a 4 inch Si wafer. Graphene flakes were obtained from natural graphite crystals (Natural Kish Graphite, Grade 300, Graphene Supermarket) and hBN flakes were obtained from hBN crystals (HQ graphene). Graphite crystals were mechanically exfoliated with Magic Scotch Tape and hBN crystals were exfoliated with Ultron Silicone-Free tape B (Ultron Systems, inc.). Wafers were cleaned previously with acetone followed by 2-propanol. Wafers were treated with a oxygen plasma cleaner process (P = 29.6 W, 15 sccm O_2_, P = 1100 mTorr) for 3 minutes for the exfoliation of large graphene flakes. This treatment was not necessary for hBN exfoliation. Graphite and hBN crystals deposited on the tape were attached to the silicon oxide substrate and subsequently heated to 100 °C for 2 minutes. Finally the tape is gently removed and graphene and hBN flakes are identified by optical microscope then by Raman spectroscopy.

### Raman spectroscopy

Raman spectra were taken with a Micro Raman Spectrometer-LabRAM HR Evolution using a visible laser (*λ* = 532 nm) with output power 100 mW and attenuated down to 10 mW with a spot size of 1 μm. A diffraction grating of 600 g/mm was used to provide a frequency shift resolution of 2 cm^−1^ (see supplementary material S1).

### Stacking

hBN/graphene/hBN heterostructures were obtained by dry transfer technique, similar to the one reported by Pizzocchero *et al*. in ref.^25^, using a PPC (Polypropylene carbonate) polymer (12% in anisole) and spin coated on a PDMS thick layer (2500 rpm, 60 s). PDMS was treated before in a oxygen plasma for 3 minutes (40 W, 40 sccm, 80 mTorr, 20 °C). Areas free of impurities and bubbles in the graphene heterostructures were selected by inspecting with Raman spectrometer.

### Device fabrication

A bar was defined on the graphene heterostructure with electron beam lithography (EBL), using polymethylmethacrylate (PMMA) as a mask. A standard cleaning process and a rapid thermal annealing was performed at 350 °C in Ar atmosphere for 15 minutes to remove eventual small non-visible bubbles and the PMMA remnant. The next steps were a second EBL for the contacts definition and an evaporation of Cr/Au = 5/45 nm with electron beam evaporator. Finally, a self-aligned EBL was performed to define the nanoconstriction mask. The concentration of PMMA in chlorobenzene used depends on the fabrication step: 4% for the mask of the first etching and contacts definition, 2% for the constriction definition.

### Etching and cryo-etching

The etching for the *side contacts* is obtained by a dry etching processing in induced coupled plasma (Plasma Pro Cobra 100) with SF_6_ atmosphere (40 sccm), P = 6 mTorr, P = 75 W at 20 °C. We found a typical etching rate of 2–3 nm/s.

Nanoconstrictions were defined by cryo-etching process with a third EBL step, which was performed at −110 °C in a controlled Ar/SF_6_ (10/40 sccm) atmosphere at P = 6 mtorr for 18 s, P = 75 W. The use of cryo temperatures and Ar allows us to obtain GNCs with very low roughness as shown in the SI. Ar gas, instead of the more common O_2_ gas for a cryoecthing recipe, reduces the possibility of passivation to a minimum. The etching rate of the cryoecthing recipe is 2 nm/s. A polymethylmethacrylate (PMMA) was employed as a mask on this step.

### Atomic force microscopy

The AFM measurements of the graphene NC were performed with a Nanotech AFM instrument controlled by Dulcinea system, operating in contact mode and equipped with silicon cantilevers. The images and line profiles were analyzed by Igor Pro and WSxM software^26^.

### Tight binding method

The electronic properties of graphene close to Dirac point can be accurately described by a tight-binding Hamiltonian for *π* electrons, ${\mathcal H} =-\sum _{\langle i,j\rangle }{t}_{ij}|i\rangle \langle j|$, where |*i*〉 is the atomic orbital of the *i*-th carbon atom^27^. The corresponding orbital energy level is set as the origin of energy without losing generality. Here *t*_*ij*_ stands for the hopping energy parameter between orbitals of the *i*-th and *j*-th carbon atoms and has been set to be constant between nearest-neighbor atoms (*t*_*ij*_ = 2.8 eV, see ref.^28^) and zero otherwise. Such approximation provides reliable results near the *K* and *K*^'^ points of the Brillouin zone, as already reported in ref.^29^.

Neglecting electron-phonon^30,31^ and electron-electron^32^ interactions in our calculations, we consider electrons in the fully coherent regime, hence with ballistic motion through the whole device. Combining the *quantum transmitting boundary method*, based on a finite element approximation^33^, and an *effective transfer matrix method* adapted for graphene (see ref.^34^ for technical details), the wave function in the whole sample and the transmission coefficient *τ*_*n*_(*E*) for each mode *n* at an energy *E* is calculated. These modes, also known as channels or subbands, arise from the transverse quantization due to the lateral confinement at the leads^35^. According to the Buttiker-Landauer formalism^35^, at very low temperature the electrical conductance is proportional to the transmission coefficient at the Fermi energy *E*_*F*_

2 $$G=\frac{2{e}^{2}}{h}\sum _{n}{\tau }_{n}({E}_{F}),$$

where the index *n* runs over the transverse modes. The factor of 2 accounts for the spin degeneracy whereas the valleys are considered explicitly in our tight-binding calculation of the transmission coefficient *τ*_*n*_. As the NC becomes wider, the number of transverse modes for a given energy increases and so does the conductance. But interestingly, we found that the main features are essentially the same provided that the conductance is plotted against *Wk*_*F*_, where *k*_*F*_ is the Fermi wavenumber and *W* is the NC width. This has an important practical consequence, namely we can extrapolate results to much larger systems than the ones fabricated experimentally and otherwise computationally very expensive (or even impossible) to simulate.

## Supplementary information

Suplemen tary infrormation file

## Data Availability

All data needed to evaluate the conclusions in the paper are present in the paper and/or the supplementary material. Additional data related to this paper may be requested from the authors.
