# Supplementary material for: Quantum nanoconstrictions fabricated by cryo-etching in encapsulated graphene
Source: Sci Rep. 2019 Sep 19;9:13572. doi: 10.1038/s41598-019-50098-z (PMC6753083; doi:10.1038/s41598-019-50098-z)
Supplement: Supplementary file 1 — Suplemen tary infrormation file [file 41598_2019_50098_MOESM1_ESM.pdf]

Quantum nanoconstrictions fabricated by cryo-etching in encapsulated graphene

V. Clericò[1], J. A. Delgado-Notario[1], M. Saiz-Bretín[2], A. V. Malyshev[2,3], Y. M. Meziani[1], P. Hidalgo[2], B. Méndez[2], M. Amado[1], F. Domínguez-Adame[2], and E. Diez[1,*]

[1]Group of Nanotechnology, USAL-NANOLAB, Universidad de Salamanca, E-37008 Salamanca, Spain
[2]Departamento de Física de Materiales, Universidad Complutense, E-28040 Madrid, Spain
[3]Ioffe Physical-Technical Institute, 26 Politechnicheskaya str., 194021 St. Petersburg, Russia
[*]enrisa@usal.es

Nanoconstrictions in encapsulated graphene

S1. Raman spectra

The quality of hBN/graphene/hBN heterostructures is strongly dependent on the number of blister or bubbles of air, water or hydrocarbons trapped at the interfaces. They represent a contaminant for the heterostructures and, for this reason, it is necessary to identify those areas free of blisters.[1] After a first qualitative assessment with optical microscope, we have chosen the best areas of the different heterostructures (free of bubbles) and performed micro-Raman spectroscopy measurements. Figure S1 shows the Raman spectrum of the final hBN/graphene/hBN heterostructure on a SiO_2/Si substrate. An indication of high crystalline quality in graphene is provided by the ratio between the 2D-peak and the G-peak.

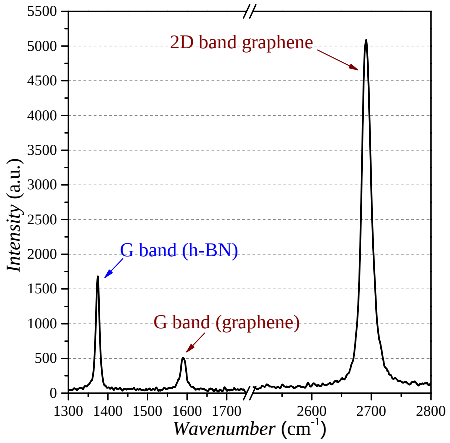

Figure S1. Typical Raman spectra for one the hBN/graphene/hBN heterostructures studied in this work. The G and 2D bands for graphene and the G band for hBN are clearly revealed and labelled accordingly.

In single layer graphene, the intensity ratio of the 2D and G peaks reaches a maximum at zero-doping, and decreases for an increasing value of doping[2]. The doping level arises mainly due to the effect of the substrate, as demonstrated by the comparison between suspended graphene with standing graphene on silicon oxide substrates[3]. The reduced intensity of the 2D peak is due to its doubly electronically resonant nature and all electron scattering processes in the resonant intermediate states decrease its intensity[4]. Therefore, this ratio provides a criterion to distinguish the doping level of graphene[5]. This criterion is still used as shown in the work of Pedrinazzi et al.[6], where the authors found a $I(2D)/I(G)$ ratio around 10 for graphene on flat poly(methylmethacrylate) layers. In our case this ratio is around 10 (see Figure S1), larger than the value observed in heterostructures obtained with similar methods[7], indicating the high quality of our hBN/graphene/hBN heterostructures. Another indication of the high quality of our heterostructures is given by the Full Width Half Maximum (FWHM) of the 2D peak as a sharper peak corresponds to a higher quality graphene flake. We found a FWHM of the 2D peak close to $18.6\,cm^{-1}$, while in graphene flakes on Si/SiO_2 substrates it is typically above $30\,cm^{-1}$ due to structural deformations that broaden the 2D

band[8].

S2. Cryo-etching process

Whereas cryo-etching was widely used in the 1980s to define Si-based structures with high aspect ratio, the appearance of a potentially cheaper method to fabricate such samples, i.e. the Bosch process where no liquid nitrogen was involved, significantly reduced the usage of cryo-etching. To our knowledge, cryo-etching has never been used to define nanostructures based on 2D materials.

In this work, we introduce for the first time a cryo-etching step to define graphene-based nanoconstrictions tailored using a commercial ICP-RIE (PlasmaPro 100 Cobra system). Such system is equipped with a cryogenic module that enables an operational temperature down to $-120\,°$C, ideal for performing cryo-etching recipes. The parameters of our cryo-etching recipe for GNC are the following: $P(\text{Rie mode}) = 75\,\text{W}$, $T = -110\,°\text{C}$, $P = 6\,\text{mTorr}$, $SF_6 = 40\,\text{sccm}$, $\text{Ar} = 10\,\text{sccm}$ for $t = 18\,\text{s}$. The etching rate of this recipe is 2 nm/s on hBN, the etching selectivity to PMMA is poor (approximately 1:3), however since the PMMA is much thicker than the top hBN flake, it is not a crucial parameter in our process. The temperature of -110°C is optimized to obtain a better vertical etching for hBN. Passivation (as well roughness of the substrate after the etching process) is reduced to the minimum thanks to the introduction of Ar gas instead of the typical O_2 used for cryo-etching of silicon. Passivation should not affect the device since the graphene is encapsulated by hBN and side contacts are deposited before the nanoconstriction is defined.. Physical etching through Ar also helps to obtain smoother edges of the NC, as is the aim of this work. The stability of the process in our machine is guaranteed by tolerances, if all the parameters do not match the set value the process is stopped. For this cryo-ecthing recipe the stability was within ± 1 degree, error on the gas flow less than 1%, and pressure of the process range less than 1 mTorr. Apart of these parameters, an important value to check the repeatability of our process is the average direct current offset of the wafer electrode from zero (earth potential), DC bias (234 V) and the power reflected (1 W).

Figure S2 shows the SEM images of NCs etched on hBN with (a) the cryo-etching technique and (b) the standard ICP-RIE procedure. The upper panels display the original SEM micrographs while the lower part coloured SEM images that help to visualize the borders of the constrictions. The higher roughness of the second image is evident at the edges, in comparison with the very well defined cryo-etched structure [1].

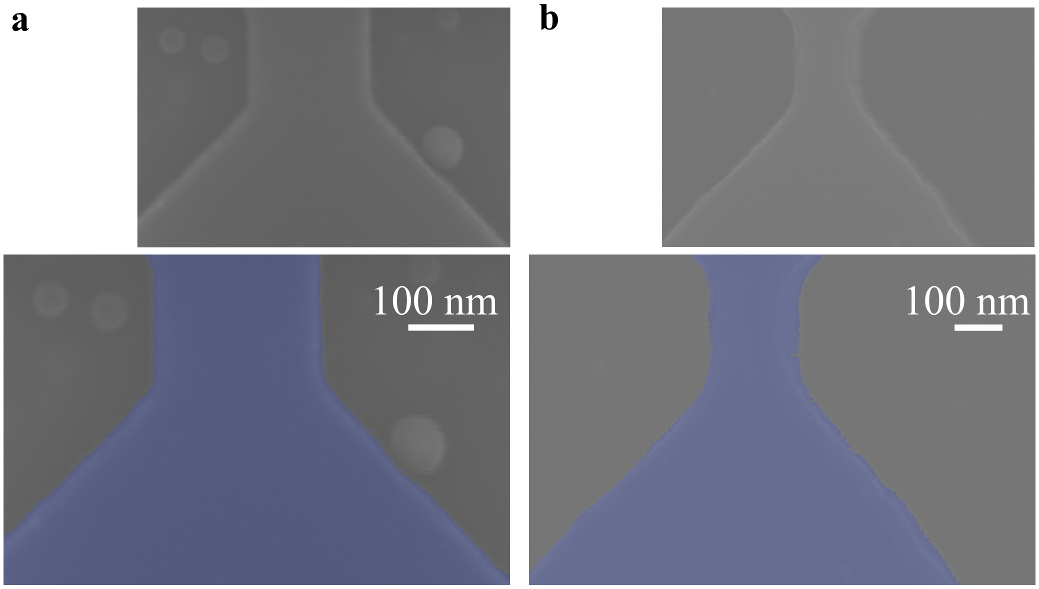

Figure S2. SEM images of NCs etched on hBN with (a) the cryo-etching technique and (b) the standard ICP-RIE procedure. The upper panel of each column shows the original SEM images in grey scale and the lower panels show a false color image to highlight the edge roughness.

[1] Three bubbles appear in the cryoetched structure, they are blisters of air trapped into the original heterostructures and in this case they are transferred to the substrate after the etching process. As the bubbles are out of the area of the device, they do not affect the device.

S3. Mobility and electron mean free path

In Figure S3a, the 4-probe resistance of encapsulated graphene versus the charge density is shown at room temperature, before constriction definition. The carrier density n is related to the gate voltage V_g as follows

$$n = \frac{C_{\mathrm{ox}}}{e}\left(V_g - V_g^*\right) , \tag{1}$$

where C_{ox} is the capacitance per unit area, $V_g - V_g^*$ is the gate voltage with respect to the charge neutrality point and e the elementary charge.

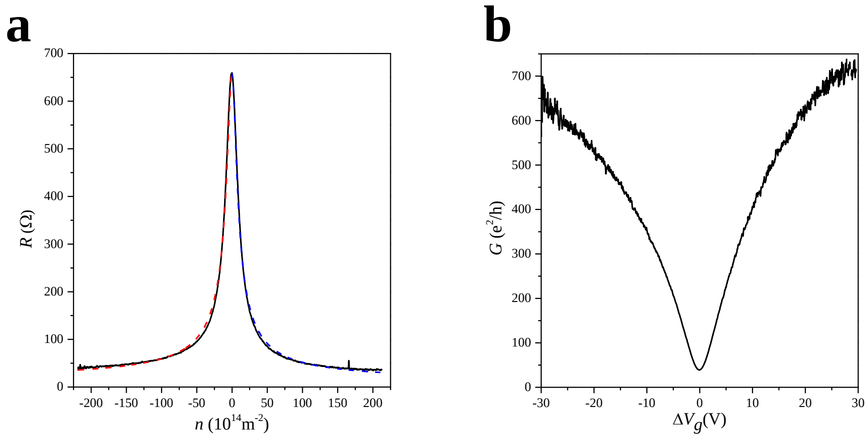

Figure S3. (a) 4-probes resistance of a graphene encapsulated device (before constriction definition) in function of the charge density n at room temperature. The mobility is estimated from Equation (2) for holes (red dashed line) and electron (blue dashed line). (b) From the resistance we calculate the conductance as $G = 1/R$. G is plotted in units of e^2/h as a function of the gate voltage with respect to the charge neutrality point $\Delta V_g = V_g - V_g^*$.

According the Ref. [9], the relation of the resistance R and the mobility μ is given by

$$R = \frac{N_{\mathrm{sq}}}{e\mu\sqrt{n_0^2 + n^2}} , \tag{2}$$

where N_{sq} is the number of squares in a graphene channel, that is, the length over the width, μ is the mobility and n_0 is the residual doping. The estimated mobility is $142\,000\,\mathrm{cm^2\,V^{-1}\,s^{-1}}$ for holes (red dashed line) and $152\,000\,\mathrm{cm^2\,V^{-1}\,s^{-1}}$ for electrons (blue dashed line). From the mobility we can determine the elastic free mean path $l = \mu\hbar/e\sqrt{n\pi}$. For an electron density $n \approx 1\times10^{12}\,\mathrm{cm^{-2}}$ we obtain a value of l larger than $1.5\,\mu\mathrm{m}$, that is, of the order of the distance between the probes. Therefore, we can confidently assume a truly ballistic regime in the whole nanostructure.

Figure S3(b) displays the conductance at room temperature as a function of the normalized gate voltage. With the 4-probe configuration, the conductance is directly obtained from the resistance as $G = 1/R$. The high values of conductance observed in our encapsulated devices are a clear signature of a very high mobility regime.

S4. Transmission parameter of hole and electron side

In Figure S4a, the conductance of 206 nm encapsulated graphene as function of the Fermi wave number k_F for the hole side ($V_g - V_g^* < 0$) is shown. The quantization steps are clearly developed for forward and backward currents (black and red line). The small shift in the red line is due to the residual gate doping but it does not affect the slope (proportional to the transmission parameter t). A linear fit of the conductance as a function of Wk_F shown in Figures S4a and S4b leads to transmission parameters $t = 0.865$ and $t = 0.863$ for the hole and electron sides, respectively. These values are very close to each other. Furthermore, they are closer to unity than the value previously reported in Ref. [10]. The larger residual carrier density on the electron side yields a threshold Fermi wavevector $k_F^0 \approx 65\times10^6\,\mathrm{m^{-1}}$, therefore the diffusive or pseudo-diffusive[11] regime is exceeded at higher values of the back-gate voltage on the electron side.

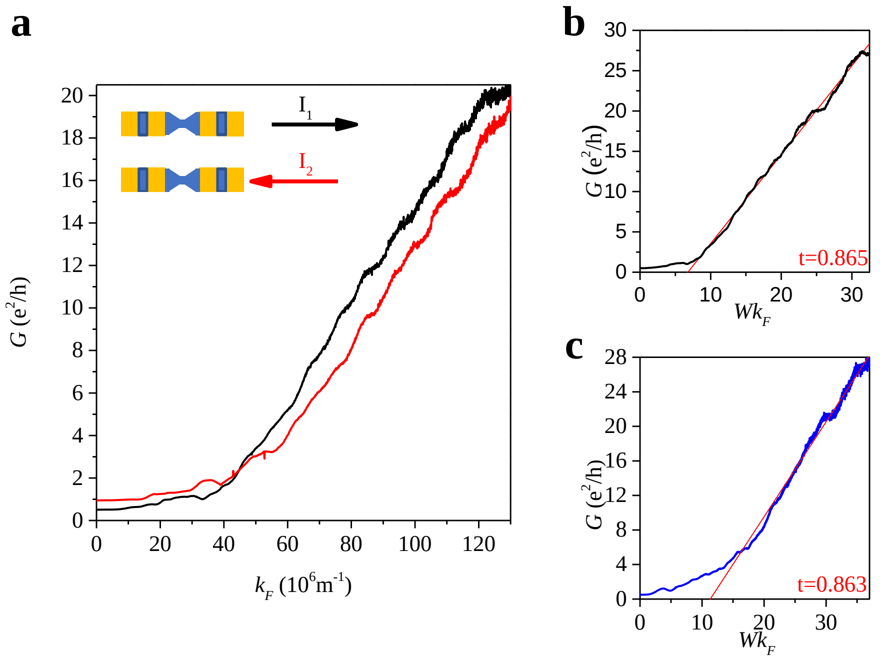

Figure S4. (a) Conductance in units of e^2/h versus the Fermi wavenumber k_F for forward and backward currents on the hole side, showing well-defined plateaus of the conductance. The transmission parameter t is obtained from the linear fitting of the conductance (in units of e^2/h) as a function of Wk_F for the (b) hole side ($t = 0.865$) and (c) electron side ($t = 0.863$). The width of the constriction is $W = 206$ nm.

Hexamethyldisilazane graphene nanoconstriction (GNC)

For the characterization of graphene nanoconstrictions (GNC) and nanoribbons (GNR) produced with a hexamethyldisilazane (HMDS) treatment, a clean piece of 1 cm^2 Si/SiO_2 substrate was dipped in a 1 : 1 solution of HMDS and acetone for at least 24 hours. After a fast cleaning (few seconds) with acetone and isopropanol, graphene is deposited onto the wafer by mechanical exfoliation, identified by optical microscopy and characterized by microRaman spectroscopy. In this way, a monolayer graphene flake is chosen and standard ohmic contacts (Ti/Au) are deposited using electron beam lithography (EBL). For the definition of the constriction/ribbon, a second step of EBL was necessary to define a PMMA mask for the etching process with inductively coupled plasma in O_2 and Ar atmosphere. The device is then suitable for transport measurements after a second immersion in HDMS for at least 24 hours.

S5. Raman spectra and electron mobility in HMDS treated graphene samples

The treatment with HMDS isolates the monolayer graphene from the substrate and, consequently, improves mobility and reduces doping effects of the substrate.[12] The presence of the HMDS polymer is first checked by MicroRaman spectroscopy. In Figure S5 we present the normalized Raman spectra of two flakes of graphene. The black line shows the Raman spectra of a monolayer graphene on Si/SiO_2 substrate and the red line corresponds to a monolayer graphene on Si/SiO_2 substrate with HMDS treatment. The intensity is normalized to unity to clearly see the difference of ratio between the 2D-peak and the G-peak. The shifts between G and 2D peak observed in Figure S5 depend on the doping level[13]. The enhancement of the ratio with the HMDS treatment (larger than a factor 3) is an indication of the improvement of the crystalline quality of the sample[14]. Using Equation (2) we estimated the mobility to be in the range 13 000 – 20 000 $cm^2\,V^{-1}\,s^{-1}$ at 4 K in graphene flakes with HMDS treatment. Using these values, the mean free path is found to be smaller than 200 nm.

S6. Measurements on a GNC with HMDS treatment

Due to the lower mean free path in these samples with respect to the encapsulated GNC, we study a GNC of 85 nm on a monolayer graphene flake with HMDS treatment. In Figure S6(a) we present the conductance in units of e^2/h as a function of Wk_F. From the linear fit (dashed red line) of the conductance versus Wk_F we found a transmission parameter $t = 0.74$ for the GNC in HMDS. The quality of the edges of the GNC are very good as shown in the insert of the Figure S6(a), where a SEM image of the same structure is shown. It is most important to highlight that the etching process of a sub-nm monolayer graphene is rather less complex than encapsulated graphene due to the dielectric nature and thickness of hBN. The main limitation of

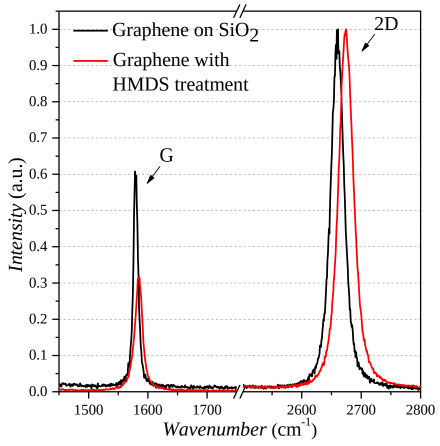

Figure S5. Normalized Raman spectra for graphene on silicon oxide (black line) and graphene with the HMDS treatment (red line).

the HMDS GNCs is the short mean free path that ensured truly ballistic transport only in small constrictions but not on the whole device. The smaller value of t in our HMDS-NC and others[15] could be related to the much lower mobility achieved. In Figure S6b we plot the conductance (black line) and the transconductance (blue line) versus Wk_F. A sequence of three quantized plateaus appear with a separation less than $2e^2/h$.

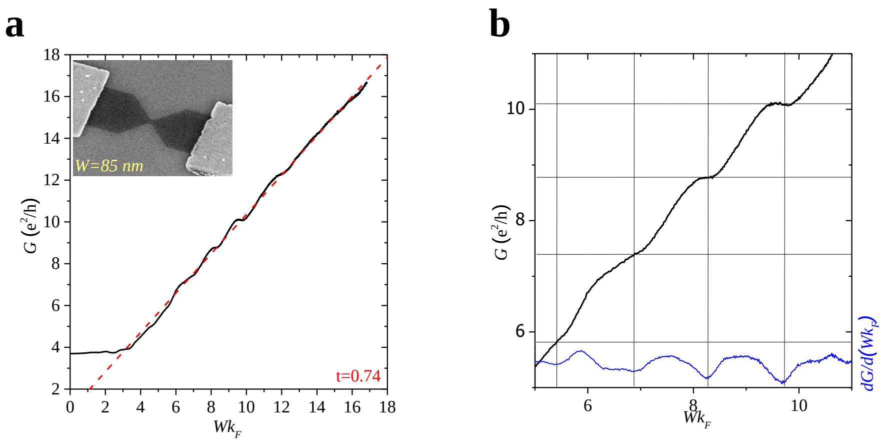

Figure S6. (a) Conductance versus Wk_F of a NC of 85 nm with HMDS treatment. (b) Enlarged view of the conductance (black line) and transconductance (blue line) versus Wk_F.

The effect of HMDS disappears after 2 days in air and less than two weeks in high vacuum at cryo-temperatures. In Figure S7, the black line shows the conductance as a function of Wk_F of the GNC ($W = 85$ nm) with HMDS treatment, while the red line is the conductance of the same GNC after the HMDS effect disappeared.

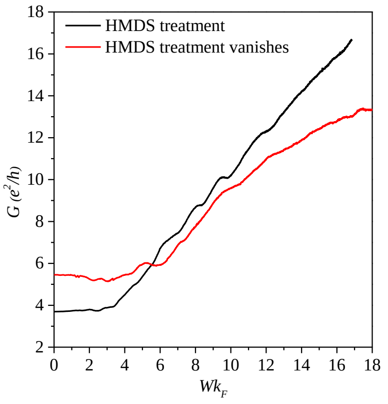

Figure S7. Conductance in units of e^2/h versus Wk_F of a HMDS GNC (black line) and without HMDS (red line).

References

1. Purdie, D. *et al.* Cleaning interfaces in layered materials heterostructures. *Nat.Comm.* **9**, 5387, DOI: 10.1038/s41467-018-07558-3 (2018).
2. Basko, D. M., Piscanec, S. & Ferrari, A. C. Electron-electron interactions and doping dependence of the two-phonon raman intensity in graphene. *Phys.Rev.B* **80**, 165413, DOI: 10.1103/PhysRevB.80.165413 (2009).
3. Berciaud, S., Ryu, S., Brus, L. E. & Heinz, T. F. Probing the intrinsic properties of exfoliated graphene: Raman spectroscopy of free-standing monolayers. *Nano Lett.* **9**, 346, DOI: 10.1021/nl8031444 (2009).
4. Basko, D. M. Theory of resonant multiphonon raman scattering in graphene. *Phys.Rev.B* **78**, 125418, DOI: 10.1103/PhysRevB.78.125418 (2008).
5. Caridad, J. M. *et al.* Effects of particle contamination and substrate interaction on the raman response of unintentionally doped graphene. *J. Appl. Phys.* **108**, 084321, DOI: 10.1063/1.3500295 (2010).
6. Pedrinazzi, P. *et al.* High-quality graphene flakes exfoliated on a flat hydrophobic polymer. *Appl. Phys. Lett.* **112**, 033101, DOI: 10.1063/1.5009168 (2018).
7. Pizzocchero, F. *et al.* The hot pick-up technique for batch assembly of van der waals heterostructures. *Nat. Comm.* **7**, 11894 (2016).
8. Neumann, C. *et al.* Raman spectroscopy as probe of nanometre-scale strain variations in graphene. *Nat. Comm.* **6**, 8429, DOI: 10.1038/ncomms9429 (2015).
9. Kim, S. *et al.* Realization of a high mobility dual-gated graphene field-effect transistor with Al_2O_3 dielectric. *Appl. Phys. Lett.* **94**, 062107, DOI: 10.1063/1.3077021 (2009).
10. Terrés, B. *et al.* Size quantization of Dirac fermions in graphene constrictions. *Nat. Commun.* **7**, 11528, DOI: 10.1038/ncomms11528 (2016).
11. Kumaravadivel, P. & Du, X. Signatures of evanescent transport in ballistic suspended graphene-superconductor junctions. *Sci. Rep.* **6**, 24274, DOI: 10.1038/srep24274 (2016).
12. Lafkioti, M. *et al.* Graphene on a hydrophobic substrate: Doping reduction and hysteresis suppression under ambient conditions. *Nano Lett.* **10**, 1149, DOI: 10.1021/nl903162a (2010).
13. Das, A. *et al.* Monitoring dopants by Raman scattering in an electrochemically top-gated graphene transistor. *Nat.Nanotech.* **3**, 210, DOI: 10.1038/nnano.2008.67 (2008).
14. Min, B. *et al.* AC-impedance spectroscopic analysis on the charge transport in CVD-grown graphene devices with chemically modified substrates. *ACS Appl. Mater. Interfaces* **8**, 27421, DOI: 10.1021/acsami.6b03705 (2016).
15. Caridad, J. M. *et al.* Conductance quantization suppression in the quantum Hall regime. *Nat. Commun.* **9**, 659, DOI: 10.1038/s41467-018-03064-8 (2018).
